# Diagnostic value of the apparent diffusion coefficient in differentiating malignant from benign endometrial lesions

**DOI:** 10.3389/fonc.2023.1109495

**Published:** 2023-04-14

**Authors:** Bojana Scepanovic, Nikola Andjelic, Ljiljana Mladenovic-Segedi, Dusko Kozic, Dusan Vuleta, Una Molnar, Olivera Nikolic

**Affiliations:** ^1^ Department of Radiological Diagnostics, Oncology Institute of Vojvodina, Sremska Kamenica, Serbia; ^2^ Department of Radiology, Faculty of Medicine, University of Novi Sad, Novi Sad, Serbia; ^3^ Department of Gynecology and Obstetrics, Faculty of Medicine, University of Novi Sad, Novi Sad, Serbia; ^4^ Department of Gynecology and Obstetrics, Clinical Center of Vojvodina, Novi Sad, Serbia; ^5^ Faculty of Sciences, University of Novi Sad, Novi Sad, Serbia; ^6^ Center for Radiology, Clinical Center of Vojvodina, Novi Sad, Serbia

**Keywords:** diffusion magnetic resonance imaging, endometrium, endometrial cancer, gynecology, pathology

## Abstract

**Introduction:**

Magnetic resonance imaging (MRI) with its innovative techniques, such as diffusion-weighted imaging (DWI) and apparent diffusion coefficient (ADC), increases the diagnostic accuracy in distinguishing between malignant and benign lesions of the endometrium. The aim of the study was MRI differentiation between malignant and benign endometrial lesions and correlation with histopathological findings with a special emphasis on quantitative analysis. An additional aim was to correlate the ADC values and histological tumor grades.

**Methods:**

The prospective study included 119 female patients with or without vaginal bleeding and pathological values of endometrial thickness, who underwent MRI examinations. According to MRI reports the patients were divided into 45 suspicious malignant and 74 suspicious benign endometrial lesions. The radiological diagnosis was compared to the histopathological evaluation, which confirmed 37 malignant lesions while the rest were benign.

**Results:**

The mean ADC value for malignant lesions was 0.761 ± 0.13×10^−3^ mm^2^/s and for benign lesions was 1.318 ± 0.20×10^−3^ mm^2^/s. The ADC values for malignant lesions were expectedly lower than those of benign lesions (p<0.001). The ADC cut-off value was 1.007×10^−3^ mm^2^/s with a sensitivity of 100%, specificity of 92.7%, a positive predictive value of 60.3%, and a negative predictive value of 100%. In comparison with the histopathological findings, the sensitivity of MRI was 100%, specificity 90.2%, positive predictive value was 82.2%, and negative predictive value was 100%. Observing the histological grades 1, 2, and 3 of endometrial carcinoma, no statistically significant differences of mean ADC values were found. The mean ADC values for histological tumor grades 1,2 and 3 were 0.803 ± 0.13×10^−3^ mm^2^/s, 0.754 ± 0.12×10^−3^ mm^2^/s and 0.728 ± 0.13×10^−3^ mm^2^/s, respectively.

**Conclusion:**

DWI and ADC values represent clinically useful tools for the differentiation between malignant and benign endometrial lesions with high sensitivity and good specificity, but the results failed to demonstrate their usefulness in differentiating histological grades of endometrial cancer.

## 1 Introduction

Endometrial cancer (EC) is ranged as the most common gynecological cancer in developed countries with an incidence of 15-25 per 100.000 women annually (1). Although EC is predominantly revealed in postmenopausal women, it is also estimated in 10-15% of premenopausal or perimenopausal women, with 2-5% of them being younger than 40 years (2, 3). The most common symptom of EC is vaginal bleeding which can often lead to early diagnosis, but in 5-10% of postmenopausal women it is asymptomatic (4–6). In these patients, EC is the cause of vaginal bleeding in about 1-14% of cases (7).

EC is divided into type I and type II. Type I is the most common and includes endometrioid adenocarcinoma accounting to 75-80% of all endometrial cancers according to literature data, while type II is more aggressive and shows a tendency to greater infiltration of the myometrium (8). The most common histological subtypes of type II are serous, clear-cell and undifferentiated EC.

In addition to EC, benign endometrial lesions are often diagnosed as causes of abnormal uterine bleeding and, among them, endometrial hyperplasia and endometrial polyps are most common (4). Both can undergo a malignant transformation in EC. Endometrial lesions are a diagnostic challenge for both gynecologists and radiologists (9). It is considered that magnetic resonance imaging (MRI) can replace the limitations of the ultrasound examination in the assessment of the nature of endometrial lesions and that, its innovative techniques, the diffusion-weighted imaging (DWI) and the apparent diffusion coefficient (ADC), can increase the diagnostic accuracy in distinguishing between malignant and benign lesions of the endometrium (10). DWI is used to display tissue characteristics based on the Brownian diffusion motion of water molecules and is useful in assessing the extension and stage of the EC, detection of metastatic lymph nodes and the assessment of the response of the EC to therapy (8, 11). ADC is joined to DWI and represents quantitative information about the diffusion of water molecules between tissue cells. In previous research ADC is considered as a reliable auxiliary parameter in differentiating between malignant and benign lesions of the endometrium and normal tissue.

In this research we focused on MRI differentiation between malignant and benign endometrial lesions in correlation with histopathological findings. A special emphasis was on the use of quantitative MRI analysis, DWI and ADC techniques, which are irreplaceable in radiological oncology. In some cases, biopsy and histopathological analysis may be limited due to the localization and size of the observed endometrial lesion, the size of the uterine cavum, congenital malformations in younger women, cervical stenosis, and the size of the obtained sample which may be insufficient for histopathological analysis. High accuracy of DWI and ADC in assessing the existence of malignant and benign lesions would contribute to the affirmation of MRI as a non-invasive method for evaluation of endometrial pathology. Another aim of the study was to correlate the ADC values and histological grade of the tumor.

## 2 Materials and methods

### 2.1 Patients

The prospective study was conducted in the period from September 2017 to June 2022 on a total sample of 143 female patients who were examined by a gynecologist due to vaginal bleeding or as a routine control and were reported to have a pathological endometrial thickness on transvaginal ultrasound (TVUS) examination. Subsequently, all patients were examined on MRI with DWI. Twenty-four patients were excluded from the study based on the exclusion criteria and the total number of patients was 119. According to the MRI results, patients were divided into two groups. The first group consisted of 45 patients with reported suspected malignant endometrial lesions, and the second group of 74 patients with reported suspected benign endometrial lesions on MRI. After MRI examination the final diagnosis was established according to histopathological evaluation, and the results were compared to MRI reports. The study was approved by the institutional ethical committee. All patients gave written informed consent to take part in this study. Inclusion criteria were: postmenopausal patients with bleeding and TVUS measured endometrial thickness greater than 5 mm, asymptomatic postmenopausal patients with TVUS measured endometrial thickness greater than 11 mm, premenopausal or perimenopausal women with abnormal uterine bleeding and TVUS measured endometrial thickness greater than 16 mm, no contraindications for MRI examination, indication set by the gynecologist to perform exploratory curettage or hysteroscopy and some of the patients were indicated for operation.

The main exclusion criteria were patients with contraindications for MRI examination, large submucosal myoma of the uterus that protruded into the lumen of the uterine cavum limiting the evaluation, endometrial changes that were unclearly displayed on the DWI and ADC map making their evaluation impossible, and the absence of subsequent histological finding.

**Figure 1:**
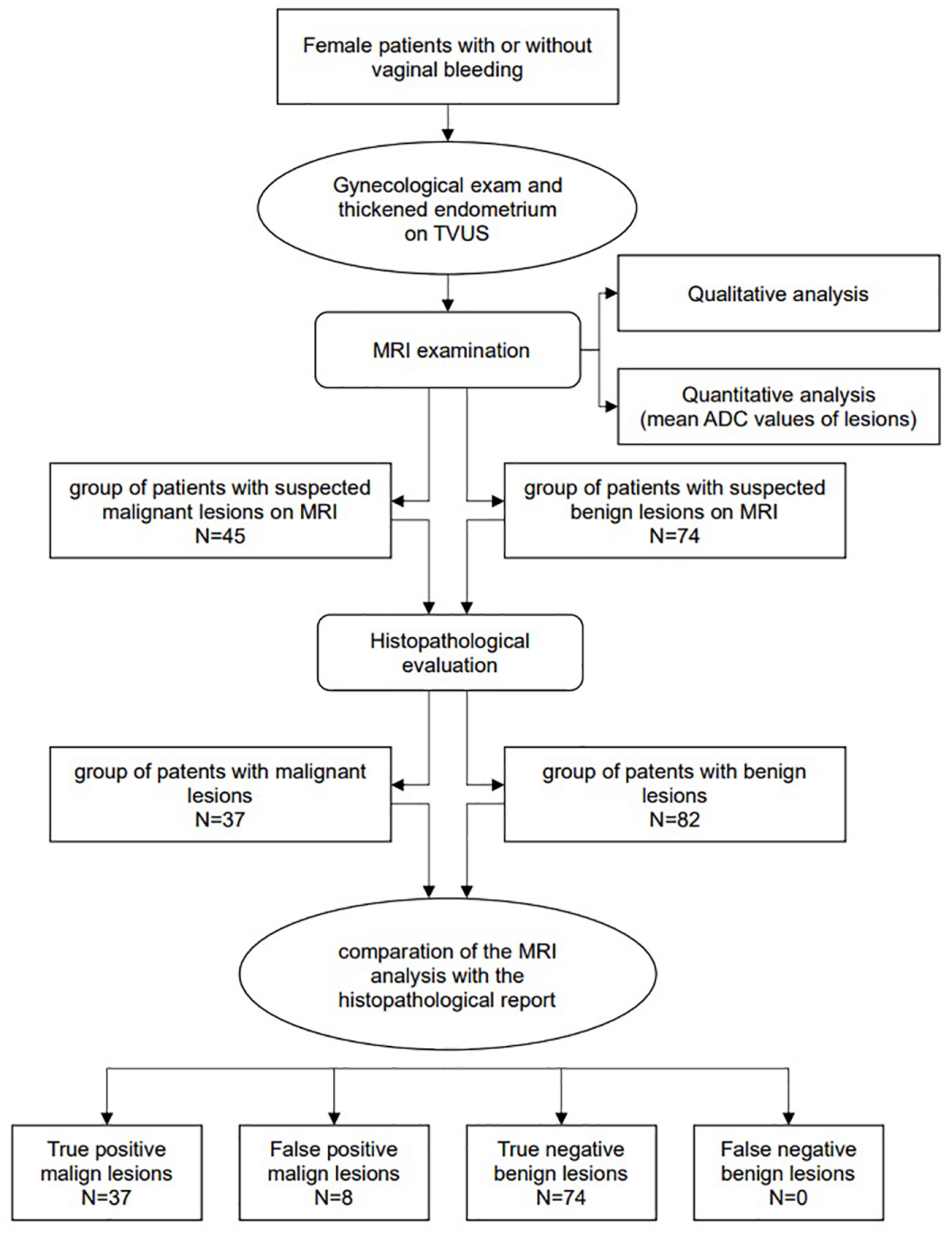
The general flowchart shows the methodological steps of the study.

The following flowchart presents methodological steps from patients’ selection phase to MRI examination and analysis (ADC measurements), histopathological evaluation and comparation between MRI and histopathology data (**Figure 1**).

### 2.2 MRI protocol

MR examination of the pelvis in all patients was performed using a 1.5 Tesla MR unit (Signa HDxt, General Electric Healthcare, Boston, MA, USA). Images were acquired with an 8-channel body array coil using the lower configuration in the supine position. The following sequences were used: T2-weighted fast relaxation fast spin-echo sequence (T2W FR FSE) sagittal, coronal and axial plane, T1-weighted fast spin-echo (T1W FSE) axial plane, T1-weighted fast spin-echo with fat suppression (T1W FSE FS) axial plane, T2-weighted fast relaxation fast spin-echo sequence (T2W FR FSE) axial oblique (perpendicular) to the uterine cavity, diffusion-weighted imaging (DWI) in the axial plane, Liver Acquisition with Volume Acquisition (LAVA) sequence in the axial plane before and after contrast administration. Apparent diffusion coefficient (ADC) maps were generated with the manufacturer’s software. The parameters of the MRI sequences are presented in **Table 1**. To prepare for the examination, patients fasted between 3 and 6 hours (except those with diabetes). Patients received an intramuscular injection of antiperistaltic agent hyoscine butylbromide (Buscopan ampoule 20 mg) to decrease artifacts due to peristaltic movements, except in cases it was contraindicated.

**Table 1:** Parameters of MRI sequences for pelvis examination.

Sequence	T2W FR FSE	T2W FR FSE	T2W FR FSE	T1W FSE	T1W FSE fs	T2W FR FSE	DWI	LAVA before and after contrast administration
Imaging plane	Sagittal	Axial	Corona	Axial	Axial	Perpendicular to the uterus	Axial	Axial
TR/TE (ms) (Repetition time/Echo time)	2760/10	2220/102	7240/102	460/min full (13.9 -37.1)	440/min full (13.1-35.0)	5560/102	10760/78.7	3.1/1.3-11.0
Matrix size	384x256	320x224	416x224	352x224	320x224	256x224	82x128	160x160
FOV (cm) (Field of view)	33x33	30x30	34x34	30x30	30x30	24x24	30x30	40x40
Slice thickness/Gap (mm)	5/1	5/1	4/1	5/1	5/1	4/1	5/1	2/-
Number of slices	30	39	34	39	39	25	39	140-248
Bandwidth (Hz)	41.67	25.00	31.25	31.25	31.25	25.00	–	62.50
NEX (Number of excitations)	2.00	3.00	2.00	2.00	2.00	6.00	–	–
b values (s/mm^2^)	–	–	–	–	–	–	0; 1200	–
Scan time (min:s)	3:30	4:54	3:59	3:49	6:23	6:13	4:40	0:33

### 2.3 Image analysis

Image analysis and measurements were done on a clinical picture archiving and communication system workstation monitor and post-processed using the General Electric Functool software package (Advantage 4.7; GE Medical Systems/Healthcare, Waukesha, WI, USA). Quantitative and qualitative analyses of the images were performed by two experienced radiologists independently, who then came to a joint conclusion. First, an evaluation of conventional and post-contrast sequences was made, which were then correlated with DWI sequence and corresponding ADC map, including both qualitative and quantitative analysis. The ADC map itself poorly shows anatomical details, so it is necessary to perform the analysis together with other MR images, which, besides DWI sequence, include high-resolution anatomical images and post-contrast images. On T2W sequence, morphological characteristics of the uterus were observed, i.e., its appearance, size, shape, as well as zonal anatomy. The corpus and cervix of the uterus were particularly observed. Then an evaluation of the appearance of the uterine cavity and the presence of any content was checked. Special emphasis was placed on the evaluation of the endometrium and its lesions as the subject of this study. The thickness of the endometrium was measured, with careful observation of its signal-morphological characteristics. On T1W sequence, the appearance of the uterus was observed, i.e., external contours, the presence of any hematometra or hemorrhagic content, the appearance of the endometrium, and lymph nodes. On T2W sequences, DWI, and post-contrast images, we evaluated the tumor invasion of the myometrium.

The characteristics of endometrial lesions were observed with an emphasis on DWI images. Lesions and areas that were suspected of malignancy showed high signal intensity on DWI images with the b value of 1200 mm^2^/s and corresponding low signal intensity on ADC maps which indicated signs of diffusion restriction. In other cases, when hyperintensity on DWI corresponded to a high signal intensity on ADC maps, lesions were suspected to be benign. The ADC values of both suspected malign and suspected benign endometrial lesions were measured manually using a circular region of interest (ROI). The ROI was manually drawn and placed on a representative region as large as possible to include only the solid parts of the endometrial lesion. We cautiously avoided areas of normal myometrium and junctional zone, cystic or necrotic areas and hemorrhagic content. To do so, the conventional and postcontrast MRI sequences were evaluated and correlated with DWI and ADC maps. The size of ROI in each case depended on the size of the endometrial lesion. The ROI was set on the T2-weighted image and was manually copied to the corresponding ADC map, whereupon ADC values were automatically calculated. Three individual ROIs were drawn at different sections of each lesion, based on which the average ADC value for each patient was calculated.

### 2.4 Histopathological evaluation

Definitive diagnoses were set based on histopathological evaluation and reports after fractionated exploratory curettage, hysteroscopy and/or surgical operation. They were made by two pathologists experienced in gynecological pathology and were in compliance with the WHO (The World Health Organization) Classification of Tumors and FIGO (International Federation of Gynecology and Obstetrics) grading of endometrial carcinoma.

### 2.5 Statistical analysis

Statistical analysis was performed using the Statistical Package for Social Science – IBM SPSS Statistics 21. Numerical variables were presented through mean values (arithmetic mean) and measures of variability (value range, standard deviation), and attributive variables were presented using frequencies and percentages. We checked a normal distribution using the Kolmogorov-Smirnov test, and appropriate tests were used in relation to that. The comparison of numerical values between two groups was performed using the Student’s t-test and Mann-Whitney test, while one-way analysis of variance (ANOVA) was used to compare values between three and more groups of data. Testing the difference in frequencies of attributive variables was performed using the χ^2^ test. ROC analysis was used to define the cut-off value of the test that gives the best ratio of specificity and sensitivity. Values of significance level p<0.05 are considered statistically significant. The results are presented in tables and figures.

## 3 Results

In our study the mean age of female patients was 63.28 ± 8.02 (range 44-82 years) and among them 112 were in postmenopausal and 7 in perimenopausal period. Mean endometrial thickness measured at TVUS examination was 15.51 ± 6.05 mm (range 7-35 mm). Vaginal bleeding was noted in 80 patients, while in 39 cases it was asymptomatic. Endometrial thickness values in patients with benign lesions were statistically significantly lower than in those with malignant lesions (t=3,850; p<0,001). Significantly more patients with malignant lesions had vaginal bleeding compared to those with benign lesions (χ^2 =^ 14,826; p<0,001).

Based on the MRI analysis, in 74 cases endometrial changes were reported as probably benign, with the mean ADC value of 1.361 ± 0.161×10^−3^ mm^2^/s (range 1.044-1.858×10^−3^ mm^2^/s) and in 45 cases they were reported as probably malign, with the mean ADC value 0.790 ± 0.14×10^−3^ mm^2^/s (range 0.542-1.059×10^−3^ mm^2^/s).

**Figure 2:**
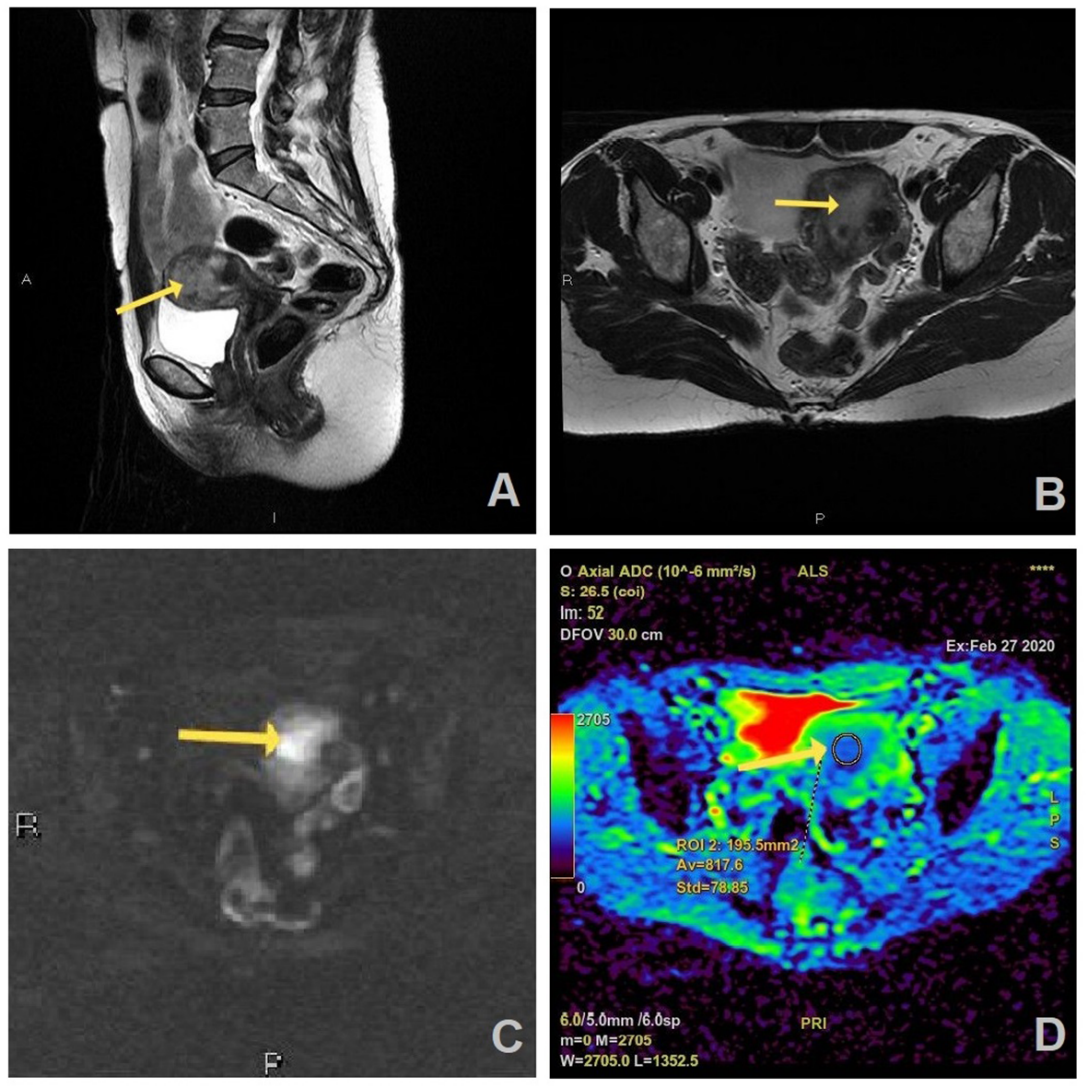
MR images of a 55-year old woman with a history of vaginal bleeding, high suspicious EC on MRI, and histopathologically proven endometrial endometrioid carcinoma, HG2 (FIGO stage II): **(A)** sagittal and **(B)** axial T2W FR FSE image shows endometrial mass (arrow) in uterine cavity which is hyperintense on axial DWI (b=1200 s/mm^2^) **(C)** and has correlation on ADC map with the measured ADC value of 0.817 × 10^−3^ mm^2^/s **(D)**.

**Figure 3:**
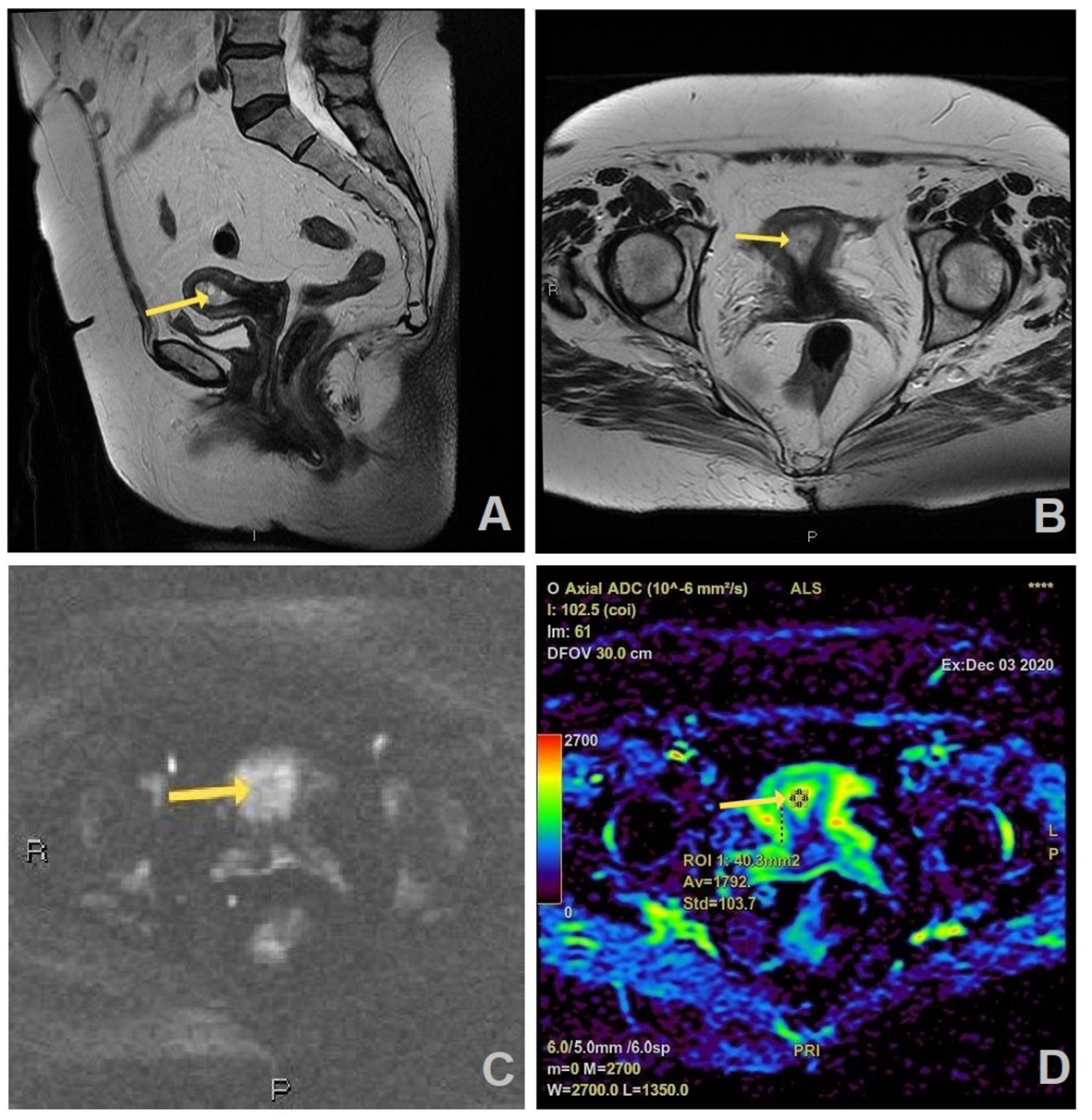
MR images of a 66-year old woman with a history of vaginal bleeding, suspicious benign lesion on MRI, and histopathologically proven endometrial hyperplasia: **(A)** sagittal and **(B)** axial T2W FR FSE image shows thickened endometrium in uterine cavity (arrow) which is slightly hyperintense on axial DWI (b=1200 s/mm^2^) **(C)** and has no correlation on ADC map with the measured ADC value of 1.792 × 10^−3^ mm^2^/s **(D)**.

**Figures 2**, **3** show the MRI appearance of the malignant and benign lesions of the endometrium from the samples of our patients.

According to histopathological reports, malignancy was confirmed in 37 out of 45 cases reported as malignant on MRI, and the rest of 82 cases were proved to be benign. The mean ADC value of confirmed malignant lesion was 0.761 ± 0.13×10^−3^ mm^2^/s (range 0.542-1.007×10^−3^ mm^2^/s), whereas the mean ADC value of benign lesions was 1.318 ± 0.20×10^−3^ mm^2^/s (range 0.756-1.858×10^−3^ mm^2^/s). The histopathological findings are summarized in **Table 2**.

**Table 2:** Histopathological diagnoses of benign and malignant endometrial lesions.

Pathohistological findings							
	Benign lesions		Malignant lesions		Total		Mean ADC (x10^−3^ mm^2^/s) ± SD
	N	%	N	%	N	%	
Endometrial endometrioid carcinoma	0	0.0	29	78.4	29	24.4	0.758 ± 0.13
Serous carcinoma	0	0.0	5	13.5	5	4.2	0.778 ± 0.09
Clear cell adenocarcinoma	0	0.0	2	5.4	2	1.7	0.660 ± 0.11
Undifferentiated carcinoma	0	0.0	1	2.7	1	0.8	0.954
Endometrial polyp	44	53.7	0	0.0	44	37.0	1.311 ± 0.20
Simple endometrial hyperplasia without atypia	30	36.6	0	0.0	30	25.2	1.344 ± 0.19
Endometrial polyp with simple endometrial hyperplasia without atypia	6	7.3	0	0.0	6	5.0	1.249 ± 0.28
Adenomyoma	2	2.4	0	0.0	2	1.7	1.292 ± 0.08
Total	82	100	37	100	119	100	

**Figure 4:**
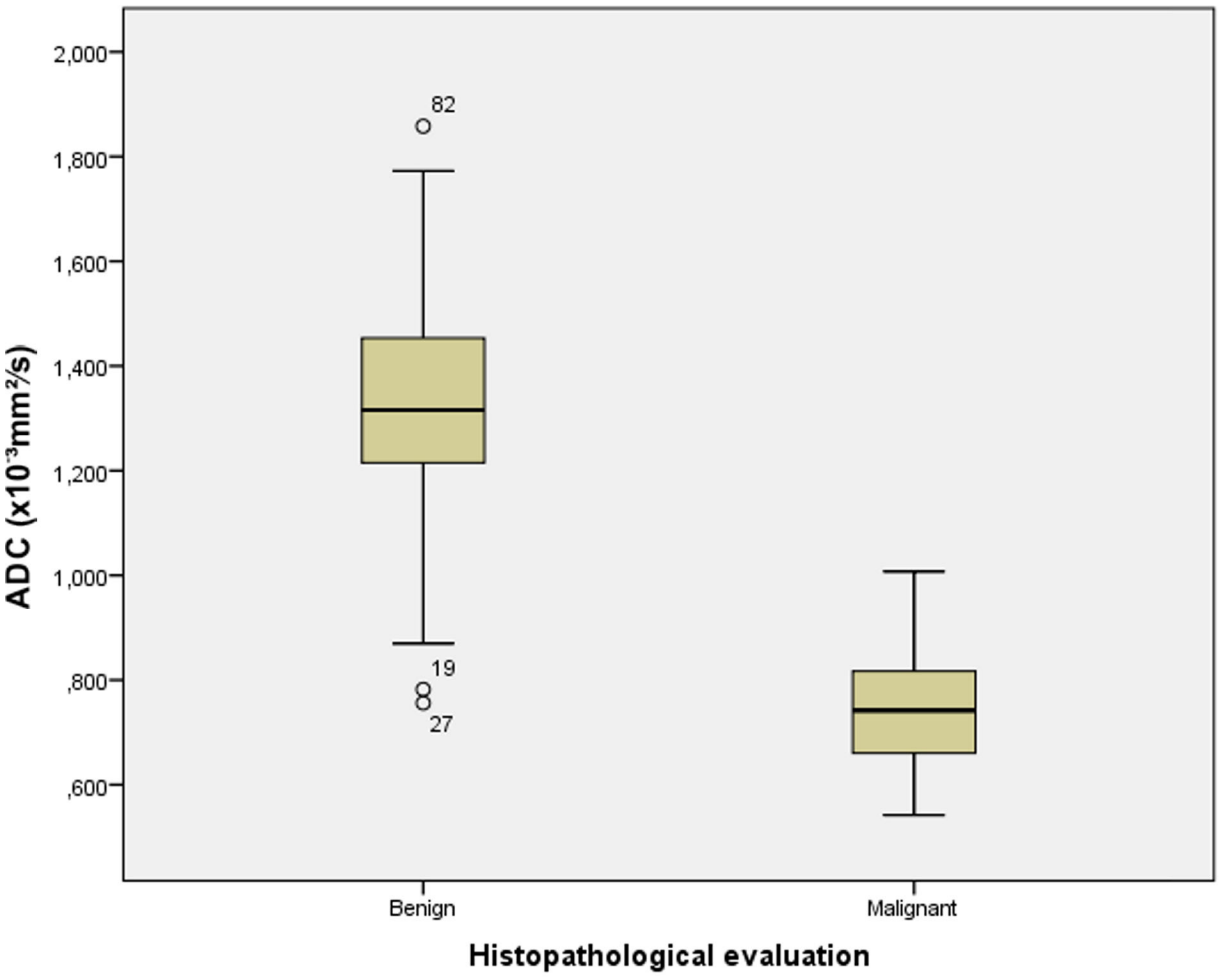
The box-and-whisker plots show the ADC values of benign and malignant endometrial lesions according to histopathological analysis.

The box-and-whisker plots presented in **Figure 4** show the distribution of ADC values between the group of benign and malignant endometrial lesions. According to the pathological findings, the mean ADC values of malignant lesions were statistically significantly lower than those of benign lesions (t=15.289; p<0.001). In the group of patients with benign lesions were some outlier values represented by circles. Two values were more than 1.5 x interquartile range below the first quartile which were the mild outliers. In our data set this were case numbers 19 and 27 with the ADC values of 0.782 x 10^-3^ mm^2^/s and 0.756 x 10^-3^ mm^2^/s, respectively. The case number 82 with the ADC value of 1.858 x 10^-3^ mm^2^/s was an extreme outlier. This value is more than 3.0 x interquartile range above the third quartile.

**Figure 5:**
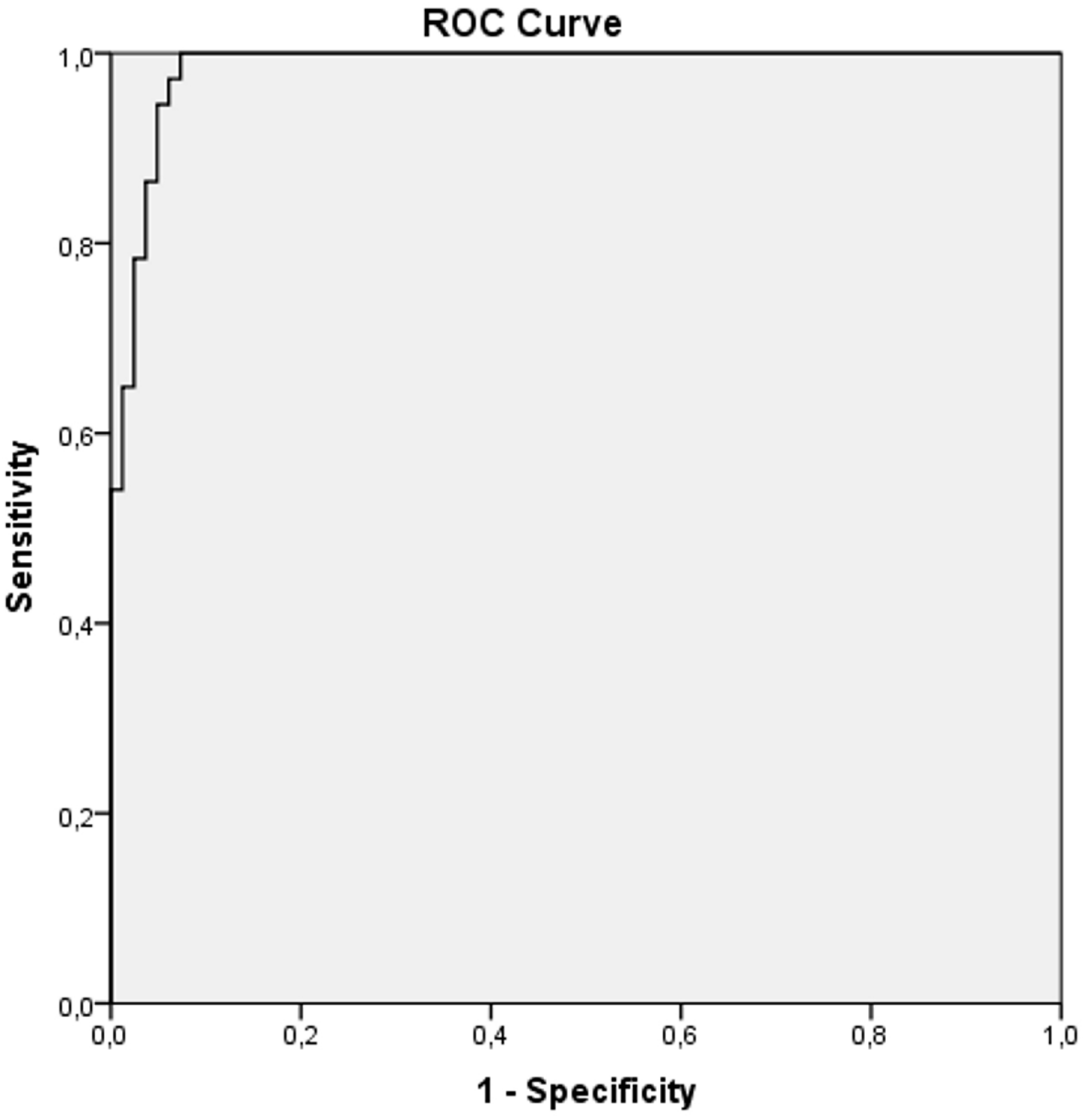
ROC curve analysis of ADC values in differentiation between malignant and benign endometrial lesions.

The results of ROC curve analysis presenting sensitivity and specificity of ADC values in differentiation between malignant and benign endometrial lesions are shown in **Figure 5**. Based on the area under the curve (AUC=0.985; CI (confidence interval) 0.968-1.000), we can see that lower ADC values predict malignant lesions with 98.5% accuracy. The ADC cut-off value was 1.007 x 10^-3^ mm^2^/s. Using this value, the sensitivity for distinguishing malignant from benign lesions was 100%, specificity was 92.7%, positive predictive value (PPV) was 60.3%, and negative predictive value (NPV) was 100%.

**Figure 6:**
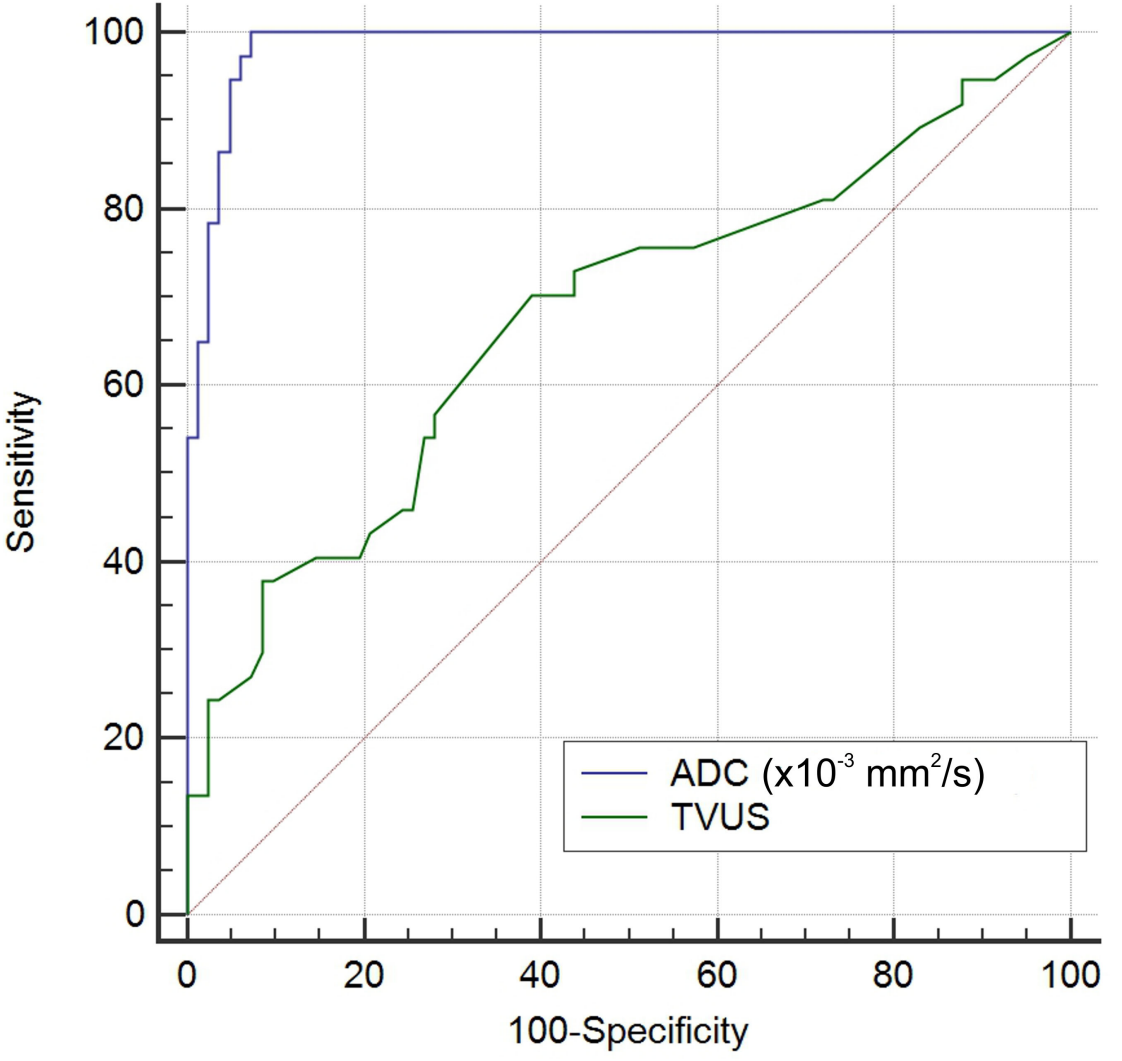
Comparison of the ROC curves of ADC values (blue curve) and endometrial thickness measured by TVUS (green curve) in differentiation between malignant and benign endometrial lesions.

**Figure 6** shows a comparison of the two ROC curves. Explanation for ROC curve analysis (marked blue) that represents sensitivity and specificity of ADC values in differentiation between malignant and benign endometrial lesions is given in previous paragraph. The second ROC curve analysis (marked green) represents sensitivity and specificity of endometrial thickness measured by TVUS in differentiation between malignant and benign endometrial lesions. Based on the area under this curve (AUC=0.676; CI 0.566-0.787), we can see that prediction for malignant lesions is 67.6% compared to benign lesions in patients with thickened endometrium. The cut-off value for endometrial thickness was 14.85mm. Using this value, the sensitivity for distinguishing malignant from benign lesions was 70.3%, specificity was 61%, PPV was 16.69%, and NPV was 94.87%. By comparing the areas under these curves, our results showed that ADC values are a statistically significantly better predictor of malignancy than TVUS-measured endometrial thickness.

By comparing the MRI findings with the findings obtained after the histopathological analysis, the sensitivity of MRI in relation to histopathological findings was 100%, specificity was 90.2%, PPV 82.2% and NPV was 100%.

**Figure 7:**
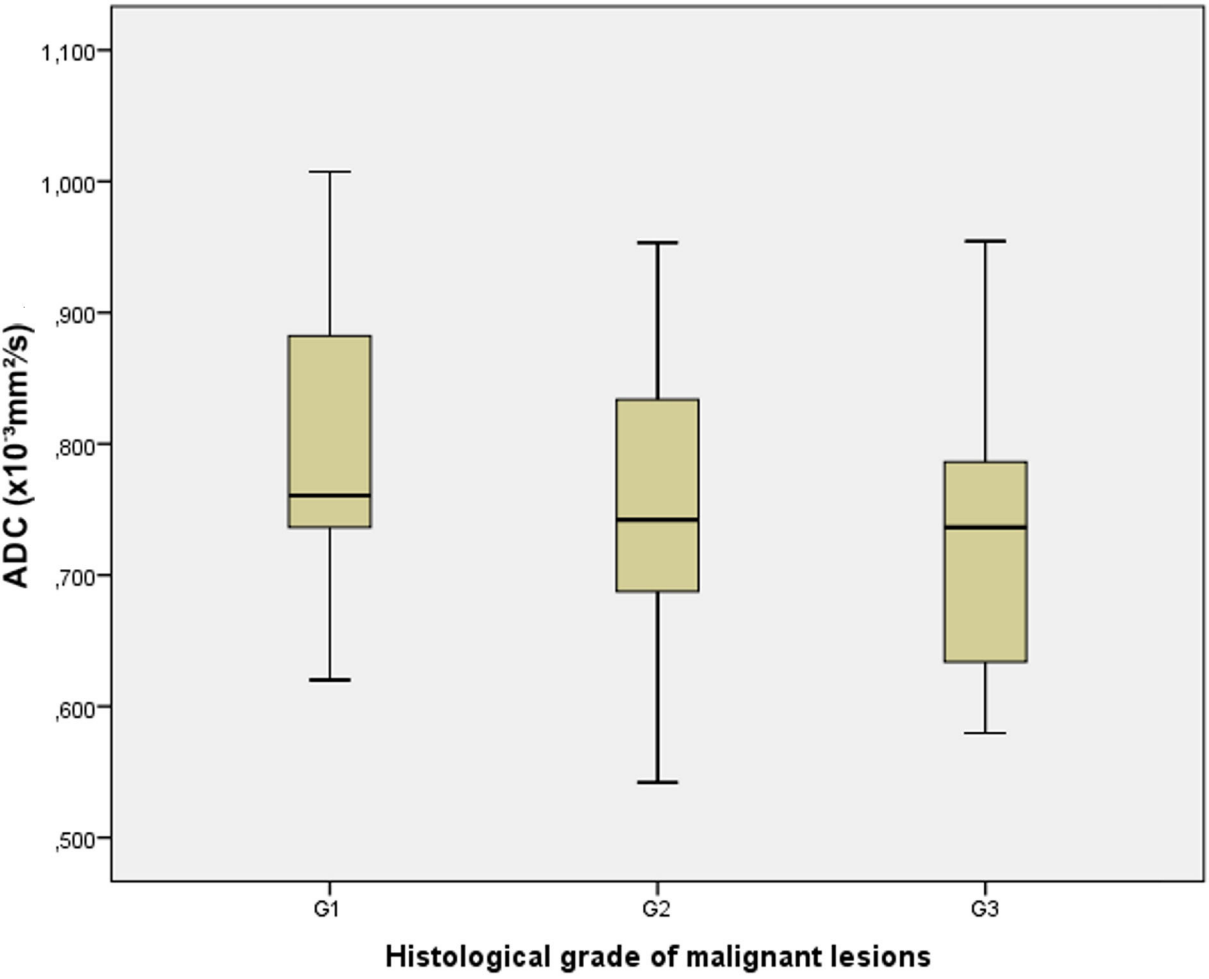
The box-and-whisker plots show the ADC values in different histological grades (G1, G2, and G3) of malignant lesions.

In the group of proved malignant lesions, the mean ADC value for histological grade 1 tumors (n=11) was 0.803 ± 0.13×10^−3^ mm^2^/s (range 0.620-1.007×10^−3^ mm^2^/s), for grade 2 (n=15) 0.754 ± 0.12×10^−3^ mm^2^/s (range 0.542-0.953×10^−3^ mm^2^/s) and for grade 3 (n=11) was 0.728 ± 0.13×10^−3^ mm^2^/s (range 0.579-0.954×10^−3^ mm^2^/s). There was no statistically significant difference (F=1.018; p=0.372) in the mean ADC values depending on the histological grade of the malignant lesions, as demonstrated in **Figure 7**.

In cases where EC was confirmed, FIGO stage was determined. According to MRI analysis and histopathological reports, the stages IA, IB, II, IIIA and IV were present (**Table 3**). Based on MRI analysis in the current study, FIGO stage IA was present in 20 patients (54.1%), IB in eight (21.6%), II in two (5.4%), III also in two patients (5.4%) and stage IV in five patients (13.5%). Referring to histopathological reports, stage IA was present in 16 patients (43.3%), IB in seven (18.9%), II in four (10.8%), III in five (13.5%) and IV stage also in five patients (13.5%). The largest number of patients had confirmed stage IA with the mean ADC value of 0.811 ± 0.13 ×10^−3^ mm^2^/s and stage IB with the mean ADC value of 0.696 ± 0.12×10^−3^ mm^2^/s. We noticed that the stages IA and IB were most represented and there was no statistically significant difference in the mean ADC values between the mentioned stages (U=29,000; p=0.071).

**Table 3:** FIGO stages of EC based on MRI analysis and histopathological reports.

FIGO stage	MR analysis			Histopathological reports		
	N	%	Mean ADC (x10^-3^mm^2^/s) ± SD	N	%	Mean ADC (x10^-3^mm^2^/s) ± SD
IA	20	54.1	0.797 ± 0.13	16	43.3	0.811 ± 0.13
IB	8	21.6	0.688 ± 0.11	7	18.9	0.696 ± 0.12
II	2	5.4	0.667 ± 0.10	4	10.8	0.748 ± 0.15
IIIA	2	5.4	0.689 ± 0.04	5	13.5	0.657 ± 0.05
IV	5	13.5	0.805 ± 0.07	5	13.5	0.804 ± 0.07

## 4 Discussion

In oncological imaging the functional DWI technique is recognized as an imaging biomarker due to its ability to detect microscopic changes in the tumor structure (12, 13). As the lack of universal, standardized range and cut-off values of the ADC for different tissue is indicated in the literature, it would be useful for each radiological center to establish specific ADC values for different tissues. In current research this has been done for EC and benign endometrial lesions based on the measurements obtained for a certain number of examined female patients (14).

Invasive diagnostic methods for obtaining tissues for histopathological analysis of the endometrium have limitations. In 2-28% of cases they cannot provide a diagnosis due to possible errors in collecting a tissue sample or obtaining an insufficient sample (10). In such cases MRI with its DWI, ADC map and ADC values may have a significant role in reaching a diagnosis. Moreover, when EC is present, they can also contribute to determining the stage of the disease and thus serve as one of the prognostic factors.

Histopathological verification of the accuracy of the DWI and ADC in the differentiation of malignant and benign changes of the endometrium would contribute to verifying the reliability of radiological MRI findings and to the affirmation of MRI as a non-invasive and preferred method in diagnostics. At the same time, the number of invasive diagnostic procedures, exploratory curettage and hysteroscopy could be reduced. It is predicted that MRI with DWI and ADC may become a method for monitoring women with risk factors for development of EC and with an initially benign endometrial lesion, which is primarily important for the early detection of EC.

In previous research the most attention has been devoted to examining the role of DWI and ADC in the differentiation of EC from various benign endometrial lesions in a more precise diagnosis of EC, as well as to the possibility of determining its histological grade (10, 15–17).

In our study the results show that there is a statistically significant difference in the ADC values for malignant versus benign endometrial lesions. The mean ADC value for malignant lesions was 0.761± 0.13×10^−3^ mm^2^/s and for benign lesions 1.318 ± 0.20×10^−3^ mm^2^/s, where the cut-off ADC value was 1.007×10^−3^ mm^2^/s. The results of most studies confirm that there is a statistically significant difference in the mean ADC value of EC in relation to benign endometrial lesions (16, 18–23). Bakir et al. and Ahmed et al. agreed that quantitative analysis with ADC map is fundamental for endometrial lesion characterization (24, 25).

Kececi et al. also evaluated the quantitative values of diffusion and showed that the ADC values of EC were significantly lower than the values of benign lesions, which was also confirmed by our results (22). Kececi and associates reported that the mean ADC value for EC was 0.94 ± 0.18×10^−3^ mm^2^/s, while our values were lower (0.761 ± 0.13×10^−3^ mm^2^/s), but there was a correlation with the cut-off value of 1.007×10^−3^ mm^2^/s (22). It should be noted that the detection of small endometrial changes and their evaluation is not always possible, and this was not the focus of our research.

Çavuşoğlu et al. conducted MRI examination also using 1.5 Tesla and made evaluation on DWI obtained with the b value of 0 and 1000 s/mm^2^. In our study DWI sequence was obtained using b values 0 and 1200 s/mm^2^. The authors also reported that the mean ADC values of EC were significantly lower (0.88 ± 0.10×10^−3^ mm^2^/s) than those of benign lesions with the calculated cut-off value of 1.18×10^−3^ mm^2^/s (26). The mean ADC value for benign lesion according to their results was 1.78 ± 0.27×10^−3^ mm^2^/s, which is higher compared with our mean ADC values.

Based on the calculated ADC cut-off value of 1.007×10^−3^ mm^2^/s on MRI examination, 45 cases in our research were diagnosed as probably malignant endometrial lesions, while 74 cases were identified as benign endometrial lesions. The sensitivity was 100%, specificity 92.7%, PPV 60.3% and NPV was 100%. Based on histopathological findings, malignant endometrial lesions were confirmed in 37 cases, while the rest were benign. MRI analysis had a sensitivity of 100%, specificity 90.2%, PPV 82.2% and NPV was 100% in relation to histopathological findings.

In the work of Moharamzad et al. where the results of eleven studies were summarized, the sensitivity ranged from 80 to 100% and the specificity was between 75 and 100%, while the cut-off values were in the range from 0.90 to 1.20×10^−3^ mm^2^/s. The highest sensitivity (100%) and specificity (97%) were observed in two studies at cut-off ADC values of 0.90 and 0.98×10^−3^ mm^2^/s (27).

Elsammak et al. also showed that the mean ADC values of malignant lesions were statistically significantly lower than the values of benign lesions (p<0.001), where the mean ADC values for malignant and benign lesions were 0.82 ± 1.09×10^−3^ mm^2^/s and 1.44 ± 0.15×10^−3^ mm^2^/s, respectively (21). In their protocol authors used three different b values to obtain DWI: 0, 800 and 1000 s/mm^2^. Based on the calculated ADC cut-off value of 1.19×10^−3^ mm^2^/s, 16 patients were diagnosed to have malignant lesions and 26 benign lesions (21). Based on histopathological diagnosis, malignancy was present in 18 cases and benign changes in 24 cases. At the cut-off value of 1.19×10^−3^ mm^2^/s for distinguishing malignant from benign lesions, sensitivity was 88.9%, specificity 100%, PPV 100% and NPV 92% (21). In our work, the sensitivity was 100%, and the specificity was lower.

A study by Shen et al. showed that on a sample of 24 EC and 7 benign lesions of the endometrium, based on DWI analysis (b=1000 s/mm^2^) and ADC value measurements, the mean ADC values for carcinoma were 0.864 ± 0.31×10^−3^ mm^2^/s and 1.277 ± 0.22×10^−3^ mm^2^/s for benign lesions with a statistically significant difference (28).

On the basis of histopathological findings of the current study, in a total of 31.1% of cases of EC, the most common subtype was endometrial endometrioid carcinoma with the mean ADC value of 0.758 ± 0.13×10^−3^ mm^2^/s. Yan et al. also reported that the endometrial endometrioid carcinoma was most common, with the mean ADC value of 0.936 ± 0.223×10^−3^ mm^2^/s, which is higher than our recorded value. In the study by Çavuşoğlu et al. all malignant lesions were endometrioid adenocarcinomas with the mean ADC value of 0.88 ± 0.10×10^−3^ mm^2^/s (26, 29).

In the current study, benign lesions were present in 68.9% of female patients, with endometrial polyp and simple endometrial hyperplasia being the most common, as in the study of Elsammak et al. and Gharibvand et al. (17, 21). Literature data point to a risk of progression of endometrial hyperplasia to EC up to 5% for endometrial hyperplasia without cell atypia and even 30% in the case of hyperplasia with atypia (30). In women diagnosed with atypical hyperplasia of the endometrium after explorative curettage, EC may also coexist, which is diagnosed later based on postoperative histopathological findings (31). In the examined sample, two patients had altered endometrium suspicious for EC, based on MRI findings, and endometrial hyperplasia with atypia was diagnosed on the histopathological reports after explorative curettage. The post-operative histopathological reports definitively confirmed the diagnosis of EC. In their study Natarajan et al. have shown that MRI has a potential diagnostic value for identifying a concurrent malignancy or malignant transformation in patients with endometrial hyperplasia with atypia (32).

Important prognostic factors for EC are the histological subtype of tumor, histological grade, stage, the depth of myometrial invasion and the presence of lymphovascular invasion, among which the stage and histological grade correlate with the risk of lymph node metastasis and the patient’s prognosis (33–35). EC with a low histological grade has a lower cell density and greater movement of water molecules in the matrix and therefore tends to have higher ADC values. Conversely, EC with a higher histological grade has a higher cell density and therefore is expected to have lower ADC values (11). In previous publications on the possibility of ADC in determining the histological grade of a tumor, the results are inconsistent. Some studies have shown that there is no statistically significant correlation between the ADC value and a certain histological grade of the tumor, which is in line with our results (15, 18, 20, 24, 26, 28, 36–38). In the present study, we did not obtain a statistically significant difference in ADC values between three different histological grades of tumors that would enable their differentiation. Some authors, such as Tamai et al. showed that the ADC values of the histological grade 3 were significantly lower compared to the grade 1 (15, 39–41). There were also overlaps in ADC values between individual histological grades, as was the case with our results. The mean ADC values overlapped and were similar between the grades 2 and 3, 0.754 ± 0.12×10^−3^ mm^2^/s and 0.728 ± 0.13×10^−3^ mm^2^/s, respectively. The mean ADC value for grade 1 was 0.803 ± 0.13×10^−3^ mm^2^/s.

Yan et al. reported a statistically significant difference in the mean ADC values between grade 1 (0.921 ± 0.133×10^−3^ mm^2^/s) and grade 2 (0.968 ± 0.240×10^−3^ mm^2^/s) in relation to the value in grade 3 (0.917 ± 0.184×10^−3^ mm^2^/s) (29).

Kakkar et al. reported that the mean ADC values of endometrial cancer for histologic grades 1, 2 and 3 were 0.72 ± 0.13×10^−3^ mm^2^/s, 0.76 ± 0.17×10^−3^ mm^2^/s and 0.74 ± 0.12×10^−3^ mm^2^/s, respectively (42). There were statistically significant differences between grade 1 and grade 2. Ozturk et al. found that high-grade EC had significantly lower ADC values compared to low-grade EC (43).

DWI can be used with great diagnostic accuracy to determine the depth of tumor invasion in the myometrium of the uterus, which strongly correlates with the presence of metastases in the lymph nodes (3% with superficial myometrial invasion and 46% with deep myometrial invasion) (33, 44). This is why it is clinically important to differentiate between superficial and deep invasion of the myometrium to plan a further therapeutic approach (33, 44). In our study the largest number of patients had confirmed stage IA with the mean ADC value of 0.811 ± 0.13 ×10^−3^ mm^2^/s and stage IB with the mean ADC value of 0.696 ± 0.12×10^−3^ mm^2^/s. As for our results, no statistically significant difference in the mean ADC values was obtained between the mentioned stages, and this corroborates with the results of several earlier studies (15, 26, 45). In contrast, Husby et al. have shown that there was a statistically significant difference in the mean ADC values of ECs with deep myometrial invasion (0.75×10^−3^ mm^2^/s), which were significantly lower than the mean ADC values of tumors with superficial invasion (0.85×10^−3^ mm^2^/s) (46).

Recent articles that have attracted the interest of prestigious medical scientific journals are about the application of artificial intelligence, specifically its subfield of deep learning-based methods. The literature indicates that weakly-supervised learning-based deep learning methods using convolutional neural networks have shown significant results in image pattern recognition (47). Published articles about deep learning-based methods in MRI diagnostics of EC include staging early EC on MR, predicting myometrial invasion in patients with stage I EC, determining the depth of myometrial invasion, and identifying lesions on MR images (48–53). In a retrospective study, Urushibara et al. examined the effectiveness of a deep learning model based on using convolutional neural networks in the diagnosis of EC on MRI images, compared to the evaluation made by three radiologists (47). The research included both histopathologically confirmed EC and benign lesions. According to their results, this model showed significantly better results based on a single image of the ADC map and axial contrast-enhanced T1-weighted image in differentiating the presence of EC compared to the radiologist’s evaluation (47). Adding other types of images with different sequences improved the diagnostic value in some cases, but without a significant difference (47). The authors pointed out several limitations in their study, including the evaluation of only one selected image. In contrast, our study evaluated all sequences to accurately place the ROI in the lesion and measure the ADC value.

In our country and other developing countries, such models of deep learning methods are currently unavailable. Our method has the advantage of being widely available and simple to apply, with the possibility of implementation in routine clinical practice in the evaluation of MR images, without requiring better computer equipment. Deep learning methods are trained to perform a specific task, and they require verification, as they may have some shortcomings depending on the input data and how they were trained (54). Based on the input data, the model can learn certain characteristic parameters of the uterus region (48). In our sample of female patients, we had one case with a bicornuate uterus where we measured ADC values in the corpus with endometrial thickening, while the endometrium in the other corpus was thin. The question is whether the deep learning method can adequately recognize and evaluate certain cases, such as those with a different shape or position of the uterus or the presence of additional lesions in or around the uterus that would require additional control by a radiologist.

## 5 Conclusion

According to the results obtained in our study DWI with ADC map and measurements of ADC values represents a clinically useful tool in differentiation between malignant and benign endometrial lesions. Determination of the cut-off ADC value increases the diagnostic accuracy. The mean ADC values of malignant lesions are significantly lower than those of benign lesions and our results are in line with most of previous studies. In correlation to histopathological reports, MRI had a high sensitivity (100%) and good specificity of 90.2%. In our study population, there was no statistically significant difference in ADC values between different histological grades of tumors, and therefore grade prediction was impossible. The limitation may be due to the small number of patients with EC in the total sample. Future research on a larger sample of patients with EC could contribute to the determination of the histological grade of the tumor as an important prognostic factor. In determining FIGO stage, the most common were stages IA and IB, and there was no significant difference in the mean ADC values.

## Data availability statement

The raw data supporting the conclusions of this article will be made available by the authors, without undue reservation.

## Ethics statement

The studies involving human participants were reviewed and approved by Clinical Center of Vojvodina, Novi Sad, Serbia. The patients/participants provided their written informed consent to participate in this study. Written informed consent was obtained from the individual(s) for the publication of any potentially identifiable images or data included in this article.

## Author contributions

The authors confirm their contribution to the paper as follows: study conception and design: ON and LM-S; data collection: BS, LM-S, and DV; analysis and interpretation of results: BS, ON, NA, and UM; draft manuscript preparation: BS, NA, and DK. All authors contributed to the article and approved the submitted version.
